# Intra‐articular corticosteroids for the treatment of osteoarthritis: A systematic review and meta‐analysis on the comparison of different molecules and doses

**DOI:** 10.1002/jeo2.12060

**Published:** 2024-06-21

**Authors:** Alessandro Bensa, Manuela Salerno, Giacomo Moraca, Angelo Boffa, C. Wayne McIlwraith, Giuseppe Filardo

**Affiliations:** ^1^ Service of Orthopaedics and Traumatology, Department of Surgery EOC Lugano Switzerland; ^2^ Università della Svizzera Italiana Faculty of Biomedical Sciences Lugano Switzerland; ^3^ Applied and Translational Research (ATR) Center, IRCCS Istituto Ortopedico Rizzoli Bologna Italy; ^4^ C. Wayne McIlwraith Translational Medicine Institute Colorado State University Fort Collins Colorado USA

**Keywords:** corticosteroids, intra‐articular injections, knee, osteoarthritis, RCTs

## Abstract

**Purpose:**

The purpose of this study was to quantify and compare the clinical relevance of the different intra‐articular corticosteroids (CS) effects in vivo for osteoarthritis (OA) treatment.

**Methods:**

The search was conducted on PubMed, Cochrane, and Web of Science in October 2023. The PRISMA guidelines were used. Inclusion criteria: animal or human randomized controlled trials (RCTs), English language and no time limitation, on the comparison of different intra‐articular CS for OA treatment. The articles' quality was assessed using the Cochrane RoB2 and GRADE guidelines for human RCTs, and SYRCLE's tool for animal RCTs.

**Results:**

Eighteen RCTs were selected (16 human and 2 animal studies), including 1577 patients (1837 joints) and 31 animals (51 joints). The CS used were triamcinolone (14 human and 2 animal studies), methylprednisolone (7 human and 1 animal study), betamethasone (3 human studies) and dexamethasone (1 human study). All studies addressed knee OA except for three human and one animal study. A meta‐analysis was performed on the comparison of methylprednisolone and triamcinolone in humans with knee OA analysing VAS pain at very short‐ (≤2 weeks), short‐ (>2 and ≤4 weeks), mid‐ (>4 and ≤8 weeks), long‐ (>8 and ≤ 12 weeks), and very long‐term (>12 and ≤24 weeks). Triamcinolone showed better post‐injection values compared to methylprednisolone at very short‐term (*p* = 0.028). No difference in terms of VAS improvement was observed at any follow‐up.

**Conclusions:**

The available preclinical and clinical literature provides limited evidence on the comparison of different CS, hindering the possibility of determining the best CS approach in terms of molecule and dose for the intra‐articular injection of OA joints.

**Level of Evidence:**

Level I.

**Abbreviations**
AAOS: American Academy of Orthopaedic Surgeons
ACR: American College of Rheumatology
ADP: average daily pain
BMI: body mass index
CBPI: Canine Brief Pain Inventory
CI: confidence interval
CS: corticosteroids
ESCEO: European Society for Clinical and Economic Aspects of Osteoporosis, Osteoarthritis and Musculoskeletal Diseases
GRADE: Grading of Recommendations Assessment, Development and Evaluation
HVAS: Hudson Visual Analogue Scale
KL: Kellgren–Lawrence
KOOS: Knee Injury and Osteoarthritis Outcome Score
MD: mean difference
OA: osteoarthritis
OARSI: Osteoarthritis Research Society International Guidelines
OMERACT‐OARSI: Outcome Measures in Rheumatology Clinical Trials and Osteoarthritis Research Society International
PRISMA: Preferred Reporting Items for Systematic Reviews and Meta‐Analysis
RCT: randomized controlled trial
SYRCLE: Systematic Review Centre for Laboratory animal Experimentation
VAS: visual analogue scale
WOMAC: Western Ontario and McMaster Universities Arthritis Index

## INTRODUCTION

Osteoarthritis (OA) is one of the most common joint diseases and represents a major cause of pain and disability in older adults [36, 37, 49], with a heavy burden on healthcare systems worldwide [18, 44, 45, 68]. Once thought to be a predominantly degenerative joint disease, OA is now recognized to present a key inflammatory component in its pathogenesis involving synovial membrane alterations and the release of pro‐inflammatory cytokines in the joint environment [2, 8, 29, 35, 60]. These elements induce chondrocytes to produce degradative enzymes of the extracellular matrix, thus affecting the articular surface [14]. In this scenario, injections of intra‐articular corticosteroids (CS), such as betamethasone, dexamethasone, methylprednisolone, and triamcinolone have been proposed as a therapeutic approach for OA management, relying on their anti‐inflammatory and analgesic properties [7, 17, 31, 55, 63].

CS act directly on nuclear steroid receptors, interrupting the inflammatory and immune OA cascade at several levels, thereby reducing pro‐inflammatory and pain mediators [26, 52]. The benefits observed in animal models and in the clinical setting have led to the widespread use of CS injections in clinical practice, and this approach has been shown to provide pain relief and joint function improvement in OA joints [4, 5, 31, 42, 43, 47]. Scientific societies and healthcare organizations endorsed the use of CS injections as part of a comprehensive treatment plan for OA. The Osteoarthritis Research Society International Guidelines (OARSI) [3], the American College of Rheumatology (ACR) [34], the European Society for Clinical and Economic Aspects of Osteoporosis, Osteoarthritis and Musculoskeletal Diseases (ESCEO) [11], and the American Academy of Orthopaedic Surgeons (AAOS) [10] have all included intra‐articular CS injections among the options to manage OA joints. However, the guidelines currently lack a recommendation on the best CS type and dose. Despite the longstanding and widespread use of intra‐articular CS injections, the most suitable CS molecule and dose to address OA in clinical practice remain debated.

The aim of this systematic review and meta‐analysis was to analyse all available animal studies and clinical trials on the comparison of different intra‐articular CS types and doses, to identify the most suitable approach for the intra‐articular treatment of OA.

## MATERIALS AND METHODS

### Literature search and article selection

A systematic review of the literature was performed on the comparison of different intra‐articular CS types and doses for the treatment of OA. The study was registered on the International Prospective Register of Systematic Reviews (PROSPERO registration number CRD42023466164). A literature search was conducted on three electronic databases (PubMed, Cochrane, and Web of Science) on 30 October 2023, with no time limitation and without any filters, using the following string: (steroid* OR corticosteroid* OR glucocorticoid* OR cortisone OR hydrocortisone OR prednisolone OR prednisone OR methylprednisolone OR triamcinolone OR dexamethasone OR betamethasone OR fludrocortisone OR deoxycorticosterone) AND (inject* OR intra‐articular* OR infiltrat*) AND (osteoarthritis OR OA).

**Figure 1:**
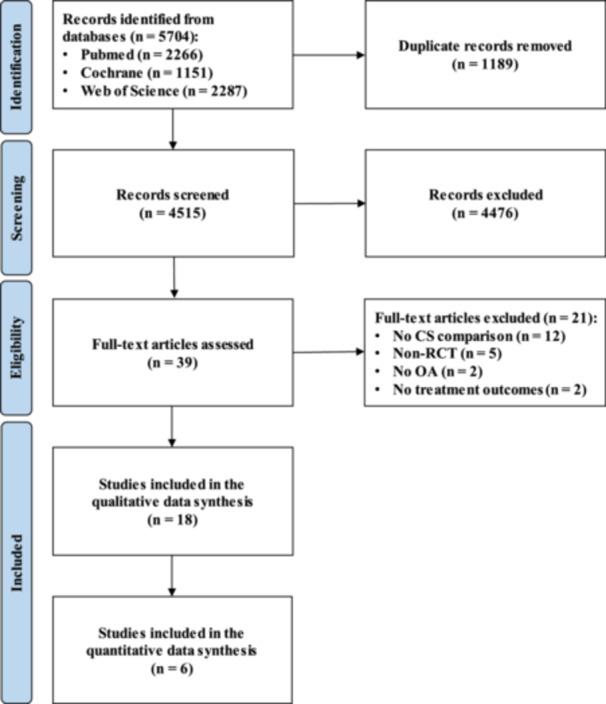
PRISMA flowchart of the study selection process. CS, corticosteroids; OA, osteoarthritis; PRISMA, Preferred Reporting Items for Systematic Reviews and Meta‐Analysis; RCT, randomized controlled trial.

According to the Preferred Reporting Items for Systematic Reviews and Meta‐Analysis (PRISMA) guidelines [46], the article selection and data extraction processes were conducted separately by two authors (A.Be. and M.S.). After the removal of duplicates, the initial title and abstract screenings were made using the following inclusion criteria: animal or human randomized controlled trials (RCTs), written in English language and with no time limitation, on the comparison of different intra‐articular CS types and doses for the injective treatment of OA. Exclusion criteria were non‐randomized studies, articles written in other languages, systematic reviews, meta‐analyses, narrative reviews, expert opinions, preclinical in vitro studies, and studies not on OA or not reporting treatment outcomes. In the second step, the full texts of the selected articles were screened, with further exclusions according to the previously described criteria. The screening process is detailed in Figure 1. Two investigators independently reviewed each article (A.Be. and M.S.), and any discrepancies between them were resolved by discussion and consensus with a third author (A.Bo.).

### Data extraction and outcome measurement

Relevant data were independently extracted from the included studies by two authors (A.Be. and M.S.). For animal studies, the data included authors, year of publication, animal type, number of animals, sex, age, weight, joint evaluated, OA model, follow‐up time, CS type and dose, number of injections, gross morphology and histological findings, biomarkers, clinical outcomes, and adverse events. For clinical studies, the data included authors, year of publication, number of patients, sex, age, body mass index (BMI), joint evaluated, follow‐up time, CS type and dose, number of injections, Kellgren–Lawrence (KL) OA grade, clinical outcomes, and adverse events. These data were collected in a database to be analysed for the present study.

The scores to evaluate the clinical benefit of different intra‐articular CS types and doses were documented and those with at least three articles contributing to each time point evaluation were considered for the meta‐analysis. Outcome analysis was performed at five different follow‐ups, resulting in very short‐ (≤2 weeks), short‐ (>2 and ≤4 weeks), mid‐ (>4 and ≤8 weeks), long‐ (>8 and ≤12 weeks), and very long‐term (>12 and ≤24 weeks) follow‐up analyses. For each outcome, the results were analysed in light of their minimal clinically important difference (MCID) values, defined as the smallest difference perceived as important by the average patient [57].

### Assessment of risk of bias and quality of evidence

The risk of bias and quality of evidence of each article was assessed independently by two authors (A.Be. and M.S.), with disagreements resolved by consensus with a third author (A.Bo.). The risk of bias in the animal studies was assessed according to the Systematic Review Centre for Laboratory Animal Experimentation (SYRCLE) tool [28]. This tool contains 10 items related to the types of bias: selection bias, performance bias, detection bias, attrition bias, reporting bias, and other biases. All items could be judged as ‘yes’ (low risk of bias), ‘no’ (high risk of bias), and ‘unclear’ (unclear risk of bias). For the evaluation of the risk of bias in the clinical studies, the Cochrane risk‐of‐bias tool for randomized trials Version 2 (RoB 2) was used [62]. RoB 2 is structured into a fixed set of bias domains, focusing on different aspects of trial design, conduct, and reporting. Within each domain, a series of questions (signalling questions) aims to elicit information about features of the trial that are relevant to the risk of bias. A proposed judgement about the risk of bias arising from each domain is generated by an algorithm, based on answers to the signalling questions. Judgement can be ‘low’ or ‘high’ risk of bias, or can express ‘some concerns’. For each plotted outcome of the meta‐analysis, the quality of evidence was evaluated according to the Grading of Recommendations Assessment, Development and Evaluation (GRADE) guidelines. In the GRADE system, the baseline rating of RCTs is considered ‘high’. Five criteria are used to downgrade one or two steps in case of ‘serious” or ‘very serious concerns’: risk of bias in individual studies, inconsistency of results between studies, indirectness of evidence, imprecision, and publication bias. The overall quality of evidence can be graded as ‘high’, ‘moderate’, ‘low’, or ‘very low’.

### Statistical analysis

The statistical analysis and the Forest plotting were carried out according to Neyeloff et al. using the Meta XL tool for Microsoft Excel by an independent professional statistician [50]. The analysis was carried out using random effects (DerSimonian & Laird) for the weighted mean differences (MD) of continuous variables. A statistical test for heterogeneity was first conducted with the Cochran *Q* statistic and *I* ^2^ metric. The presence of significant heterogeneity was considered with *I* ^2^ ≥ 25%. When no heterogeneity was found with *I* ^2^ < 25%, a fixed effect model was used to estimate the expected values and 95% confidence intervals (CIs); otherwise, a random‐effect model was applied and an *I* ^2^ metric was evaluated for the random effect to check the correction of heterogeneity. A *p* value of 0.05 was considered significant. The comparison among groups was based on the analysis of variance of the difference between basal and follow‐up scores MD [9]. All statistical analyses were carried out with Microsoft Excel.

## RESULTS

### Article selection

**Figure 2:**
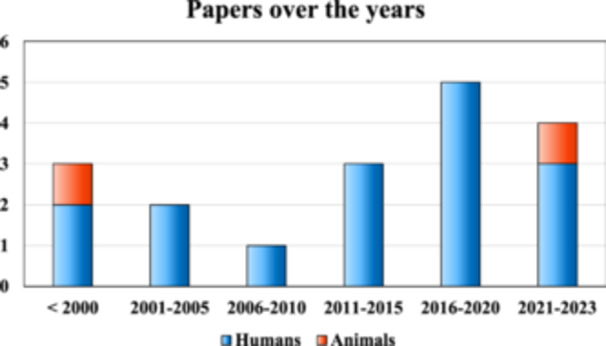
Number of randomized controlled trials published over time on the comparison between intra‐articular CS types and doses for osteoarthritis injective treatment. CS, corticosteroids.

After duplicate removal, the initial search identified 5704 records, whose abstracts were screened and selected according to the inclusion/exclusion criteria for a total of 39 articles assessed for eligibility. Twenty‐one studies were excluded after full‐text evaluation: 12 not comparing different CS, 5 non‐RCT, 2 not concerning OA, and 2 not reporting treatment outcomes. Thus, a total of 18 RCTs were included in this systematic review and meta‐analysis. Of these, two studies focused on animals (Table 1) and 16 studies on humans (Table 2). The first study comparing different CS was published in 1981, and then the publication trend increased over time, with a peak of studies in the period between 2016 and 2020 (Figure 2).

**Table 1:** Characteristics of the included animal studies.

Author	Animals	Joint	OA model	CS type	CS dose	Animals (joints) evaluated	F	M	Age	Weight (kg)
Alves 2022 [1]	Dogs	Hip	Naturally occurring	Triamcinolone acetonide	20 mg	10 (20)	5	15	6.0 ± 2.4 years	33.3 ± 4.14
				Methylprednisolone acetate	40 mg	10 (20)				
Williams 1985 [69]	Guinea pigs	Knee	Sodium iodoacetate (0.3 mg/kg) intra‐articular injection	Triamcinolone hexacetonide	0.4 mg/kg	6 (6)	0	11	32–40 weeks	0.57
				Triamcinolone hexacetonide	0.04 mg/kg	5 (5)				

Abbreviations: CS, corticosteroids; F, females; M, males; OA, osteoarthritis.

**Table 2:** Characteristics of the included clinical studies.

Author	Blinding	Joint	CS type	CS dose	Associated treatments	Patients (joints) included	Patients (joints) final follow‐up	W	M	Age (years)	BMI (kg/m^2^)	AEs
Utamawatin 2023 [66]	Double	Knee	Triamcinolone acetonide	10 mg	Lidocaine 1%, 3 mL	42	42	35	7	69.6 ± 9.2	24.9 ± 3.5	0
			Triamcinolone acetonide	40 mg	Lidocaine 1%, 3 mL	42	42	35	7	69.9 ± 11.3	25.4 ± 3.9	0
Kivitz 2022 [33]	NR	Hip	Triamcinolone acetonide extended‐release	32 mg 5 mL	NR	15	11	8	7	58.1 ± 7.6	29.3 ± 3.5	0
			Triamcinolone acetonide crystalline suspension	40 mg 5 mL	NR	15	14	5	10	60.1 ± 7.1	31.0 ± 4.4	0
Hanson 2021 [27]	NR	Shoulder	Triamcinolone acetonide extended‐release	32 mg 5 mL	NR	12	12	9	3	63.2 ± 11.4	28.9 ± 6.0	0
			Triamcinolone acetonide crystalline suspension	40 mg 1 mL	NR	13	13	6	7	61.9 ± 8.1	30.7 ± 3.6	0
Kivitz 2019 [32]	NR	Knee	Triamcinolone acetonide extended‐release	64 mg 5 mL	Microspheres	12 (24)	12 (24)	10	2	61.8 ± 6.5	33.3 ± 3.7	1
			Triamcinolone acetonide crystalline suspension	80 mg 1 mL	NR			9	3			0
Conaghan 2018 [17]	Double	Knee	Triamcinolone acetonide extended‐release	32 mg 5 mL	Microspheres	161	161	103	58	61.5 ± 9.5	30.1 ± 5.0	7
			Triamcinolone acetonide crystalline suspension	40 mg 1 mL	NR	162	161	97	64	62.3 ± 10.1	30.3 ± 4.8	4
Conaghan 2018 [16]	Double	Knee	Triamcinolone acetonide extended‐release	16 mg 5 mL	Microspheres	102	102	62	40	58.2 ± 8.3	30.6 ± 4.9	1
			Triamcinolone acetonide extended‐release	32 mg 1 mL	Microspheres	104	104	51	53	58.7 ± 8.1	31.0 ± 4.6	3
Jameel 2018 [30]	NR	Knee	Methylprednisolone acetate	40 mg 1 mL	Lidocaine 1%, 3 mL	100 (200)	NR	NR	NR	60.3 ± 2.6	27.1 ± 2.4	NR
			Triamcinolone hexacetonide	40 mg 2 mL	Lidocaine 1%, 3 mL							NR
Buyuk 2017 [12]	Double	Knee	Methylprednisolone acetate	40 mg 1 mL	NR	126 (252)	NR	101	25	68.5 ± 9.0	26.3 ± 2.6	0
			Triamcinolone hexacetonide	40 mg 2 mL	NR							0
Bodick 2015 [7]	Double	Knee	Triamcinolone acetonide extended‐release	10 mg 3 mL	Microspheres	58	56	26	32	61.5 ± 8.5	31.0 ± 4.7	1
			Triamcinolone acetonide extended‐release	40 mg 3 mL	Microspheres	59	57	31	28	60.9 ± 9.6	31.2 ± 4.4	0
			Triamcinolone acetonide extended‐release	60 mg 3 mL	Microspheres	60	59	34	26	61.9 ± 9.4	30.0 ± 4.9	0
			Triamcinolone acetonide crystalline suspension	40 mg 1 mL	NR	51	49	29	22	61.6 ± 10.1	29.8 ± 5.0	2
Lomonte 2015 [38]	Double	Knee	Methylprednisolone acetate	40 mg	NR	50	49	45	5	66.2 ± 8.2	28.5 ± 2.8	1
			Triamcinolone hexacetonide	40 mg	NR	50	46	49	1	64.8 ± 8.3	28.8 ± 2.7	0
Yavuz 2012 [71]	NR	Knee	Triamcinolone acetonide	40 mg 1 mL	NR	30	30	19	11	60 ± 5.9	NR	0
			Betamethasone disodium phosphate	3 mg 1 mL	NR	30	30	20	10	60 ± 6.3	NR	0
			Methylprednisolone acetate	40 mg 1 mL	NR	30	30	18	12	61 ± 6.3	NR	0
Wollstein 2007 [70]	Double	Knee + shoulder	Methylprednisolone acetate	40 mg 1/2 mL	Lidocaine 2%, 2 mL	46	46	49	36	65.6 ± 12.1	NR	0
			Betamethasone dipropionate + sodium phosphate	7 mg 1/2 mL	Lidocaine 2%, 2 mL	39	39					0
Pyne 2004 [53]	Double	Knee	Methylprednisolone acetate	40 mg 1 mL	NR	28	28	22	6	62.2	NR	NR
			Triamcinolone hexacetonide	20 mg 1 mL	NR	29	29	22	7	62.8	NR	NR
Bias 2001 [6]	Not blinded	Knee	Dexamethasone palmitate	4 mg 1 mL	NR	12	12	9	15	63.3 ± 5.5	NR	0
			Dexamethasone palmitate	12 mg 3 mL	NR	12	12					0
Thorpe 1985 [65]	Double	Knee	Methylprednisolone acetate	40 mg 1 mL	Lidocaine 1%, 1 mL	23 (37)	NR (34)	20	3	71.1 ± 1.3	NR	0
			Triamcinolone acetonide	10 mg 1 mL	Lidocaine 1%, 1 mL	21 (30)	NR (22)	18	3	67.2 ± 1.5	NR	0
Valtonen 1981 [67]	Single	Knee	Triamcinolone hexacetonide	20 mg 1 mL	NR	21	NR	NR	NR	66.6 ± 7.2	NR	0
			Betamethasone acetate + disodium phosphate	6 mg 1 mL	NR	21	NR	NR	NR	66.7 ± 7.5	NR	0

Abbreviations: AE, adverse event; BMI, body mass index; CS, corticosteroids; M, men; NR, not reported; W, women.

### Systematic review

#### Preclinical studies

Both animal RCTs did not report blinding details on the assessors. A total of 31 animals and 51 joints were assessed. One study analysed 11 male guinea pigs (age 32–40 weeks, mean weight 570 g) with unilateral knee OA induced by an injection of sodium iodoacetate 0.3 mg/kg and then treated with a single injection of triamcinolone hexacetonide after 24 hours. One group received a 0.4 mg/kg dose, while the other one a 0.04 mg/kg dose. The 0.4 mg/kg dose of triamcinolone hexacetonide showed superior benefits in preserving the articular surface and the pericellular proteoglycans, as well as in reducing chondrocyte loss and osteophyte formation. The other study focused on a sample of 20 dogs (25% females and 75% males, mean age 6.0 ± 2.4 years, mean weight 33.3 ± 4.1 kg) with naturally occurring bilateral hip OA, for a total of 40 hip joints treated with a single CS injection. One group received 20 mg of triamcinolone acetonide, while the other one 40 mg of methylprednisolone acetate. No statistically significant difference was found between the two CS treatments on the pain results analysed in terms of Canine Brief Pain Inventory (CBPI) and Hudson Visual Analogue Scale (HVAS) at any follow‐up. None of the two studies reported any adverse event.

#### Clinical studies

Among human RCTs, nine studies were double‐blind, one study was single‐blind (assessor), one was not blinded, and five studies did not report blinding details. A total of 1576 patients (64.1% women and 35.9% men, mean age 63.3 ± 3.6 years, mean BMI 29.4 ± 2.2 kg/m^2^) and 1837 joints were analysed. Fourteen RCTs focused on knee OA, 2 on shoulder OA, and 1 on hip OA (1 study addressed both knee and shoulder OA). Different types of CS were used, including triamcinolone (14 studies, 1286 patients), methylprednisolone acetate (7 studies, 403 patients), betamethasone (3 studies, 90 patients), and dexamethasone palmitate (1 study, 24 patients). Triamcinolone was used in the acetonide form in nine studies and in the hexacetonide form in five studies. Triamcinolone acetonide was administered in the crystalline suspension formulation in eight studies and in the extended‐release formulation in six studies (five studies compared the crystalline suspension and extended‐release formulations). Betamethasone was used in the disodium phosphate form in one study, in the dipropionate + sodium phosphate form in one study, and in the acetate + disodium phosphate form in one study. The CS dose ranged from a minimum of 3 mg of betamethasone disodium phosphate to a maximum of 80 mg of triamcinolone acetonide. In all studies, a single injection of CS was administered. The injected volume was reported in 15 studies, ranging from 1 to 5 mL. Lidocaine (1–3 mL) was reported as an associated treatment in four studies, and in all cases, it was mixed with the CS in the same syringe and administered as a single injection.

The most used scores for clinical outcome evaluation were Visual Analogue Scale (VAS) for pain in 12 studies, Western Ontario and McMaster Universities Arthritis Index (WOMAC) in 7 studies, Knee Injury and Osteoarthritis Outcome Score (KOOS) in 2 studies, Outcome Measures in Rheumatology Clinical Trials and Osteoarthritis Research Society International (OMERACT‐OARSI) criteria of response in 2 studies, Lequesne index in 2 studies, and average daily pain (ADP) in 1 study. Moreover, two studies analysed the index of activity and the knee pain and joint tenderness, respectively, evaluating them as none, mild, moderate, or severe.

A total of 457 adverse events out of 1837 injections (24.9%) were reported across all the included clinical studies. The great majority (437, 95.6%) were mild in nature (i.e., arthralgia, headache, and back pain), while 20 adverse events (4.4%) were considered more serious. These more serious adverse events were reported in five RCTs, while nine RCTs reported no serious adverse events in any of the study groups, and two RCTs did not report data on adverse events. Nineteen of these 20 more serious adverse events were reported in patients receiving triamcinolone acetonide (1098 joints studied, 1.8%), 13 with the extended‐release formulation (583 joints studied, 2.2%) and 6 with the crystalline suspension formulation (515 joints studied, 1.2%), and none was classified as treatment‐related. Only 1 serious adverse event was reported in the methylprednisolone group (417 joints studied, 0.2%) and was considered treatment‐related (post‐injection arthritis requiring arthrocentesis and non‐steroidal anti‐inflammatory drugs). Table 3 reports the results of the clinical studies included in the systematic review.

**Table 3:** Results of the included clinical studies.

Author	CS type	Patients (joints) included	Outcomes	Final follow‐up (weeks)	Overall authors' conclusions
Utamawatin 2023 [66]	Triamcinolone acetonide	42	VAS, WOMAC, EQ‐5D, KOOS, AEs	12	The 10 mg of triamcinolone acetonide is non‐inferior to 40 mg triamcinolone acetonide in improving pain in patients with symptomatic knee OA.
	Triamcinolone acetonide	42			
Kivitz 2022 [33]	Triamcinolone acetonide extended‐release	15	Plasma triamcinolone acetonide concentration, AEs	12	Systemic triamcinolone acetonide exposure was markedly lower in triamcinolone acetonide extended‐release‐treated patients. The safety profiles were comparable.
	Triamcinolone acetonide crystalline suspension	15			
Hanson 2021 [27]	Triamcinolone acetonide extended‐release	12	Plasma triamcinolone acetonide concentration, AEs	12	Lower peak and systemic triamcinolone acetonide exposure following triamcinolone acetonide extended‐release.
	Triamcinolone acetonide crystalline suspension	13			
Kivitz 2019 [32]	Triamcinolone acetonide extended‐release	12 (24)	Plasma triamcinolone acetonide concentration, AEs	6	Plasma triamcinolone acetonide concentrations were lower after bilateral triamcinolone acetonide extended‐release.
	Triamcinolone acetonide crystalline suspension				
Conaghan 2018 [17]	Triamcinolone acetonide extended‐release	161	WOMAC, KOOS‐QOL, ADP, AEs	24	ADP improvements were not significant, while exploratory analyses of WOMAC‐A, ‐B and ‐C and KOOS‐QOL subscales favoured triamcinolone acetonide extended‐release.
	Triamcinolone acetonide crystalline suspension	162			
Conaghan 2018 [16]	Triamcinolone acetonide extended‐release	102	VAS, WOMAC, AEs	24	A dose‐response effect in the duration of maximal analgesic effect was evident, with the 32 mg dose providing increased therapeutic benefit relative to the 16 mg dose.
	Triamcinolone acetonide extended‐release	104			
Jameel 2018 [30]	Methylprednisolone acetate	100 (200)	VAS, WOMAC, AEs	24	There is no significant difference between methylprednisolone acetate and triamcinolone hexacetonide, both are equally effective.
	Triamcinolone hexacetonide				
Buyuk 2017 [12]	Methylprednisolone acetate	126 (252)	VAS, WOMAC, AEs	24	Methylprednisolone acetate and triamcinolone hexacetonide have similar efficacy in relieving pain and improving function.
	Triamcinolone hexacetonide				
Bodick 2015 [7]	Triamcinolone acetonide extended‐release	58	VAS, WOMAC, AEs	12	Intra‐articular injection of triamcinolone acetonide extended‐release provided a clinically relevant improvement in pain relief in patients with knee osteoarthritis relative to triamcinolone acetonide crystalline suspension.
	Triamcinolone acetonide extended‐release	59			
	Triamcinolone acetonide extended‐release	60			
	Triamcinolone acetonide crystalline suspension	51			
Lomonte 2015 [38]	Methylprednisolone acetate	50	VAS, WOMAC, Lesquesne index, OMERACT‐OARSI, AEs	24	Both intra‐articular therapies are equally effective, and improvement in pain and function can be sustained for up to 24 weeks.
	Triamcinolone hexacetonide	50			
Yavuz 2012 [71]	Triamcinolone acetonide	30	VAS, AEs	12	Methylprednisolone acetate was a statistically more effective analgesic as compared to the other agents until the sixth week.
	Betamethasone disodium phosphate	30			
	Methylprednisolone acetate	30			
Wollstein 2007 [70]	Methylprednisolone acetate	46	VAS, AEs	3	This study does not support a difference in short‐term pain between preparations.
	Betamethasone dipropionate + sodium phosphate	39			
Pyne 2004 [53]	Methylprednisolone acetate	28	VAS, Lequesne index	8	Triamcinolone hexacetonide acts quicker and is better than methylprednisolone acetate at Week 3, but its effect is lost by Week 8. Methylprednisolone acetate has a slower onset of action and continues to show benefit at Week 8.
	Triamcinolone hexacetonide	29			
Bias 2001 [6]	Dexamethasone palmitate	12	VAS, AEs	4	Dexamethasone palmitate exhibits short‐ to medium‐term sustained release properties, together with good efficacy and very good tolerability.
	Dexamethasone palmitate	12			
Thorpe 1985 [65]	Methylprednisolone acetate	23 (37)	VAS, AEs	20	10 mg of triamcinolone acetonide has been shown to be equivalent to 40 mg methylprednisolone acetate. Injections of both corticosteroids were well tolerated and no adverse local or systemic effects were observed.
	Triamcinolone acetonide	21 (30)			
Valtonen 1981 [67]	Triamcinolone hexacetonide	21	Local pain and tenderness, AEs	24	Triamcinolone hexacetonide is a preferable drug when a long‐term effect is needed in the treatment of osteoarthrosis with concomitant inflammation.
	Betamethasone acetate + disodium phosphate	21			

Abbreviations: ADP, average daily pain; AE, adverse event; EQ‐5D, EuroQol‐5 Dimensions; KOOS, Knee Injury and Osteoarthritis Outcome Score; OA, osteoarthritis; OMERACT‐OARSI, Outcome Measures in Rheumatology Clinical Trials and Osteoarthritis Research Society International; QOL, quality of life; VAS, visual analogue scale for pain; WOMAC, Western Ontario and McMaster Universities Arthritis Index.

### Meta‐analysis

**Figure 3:**
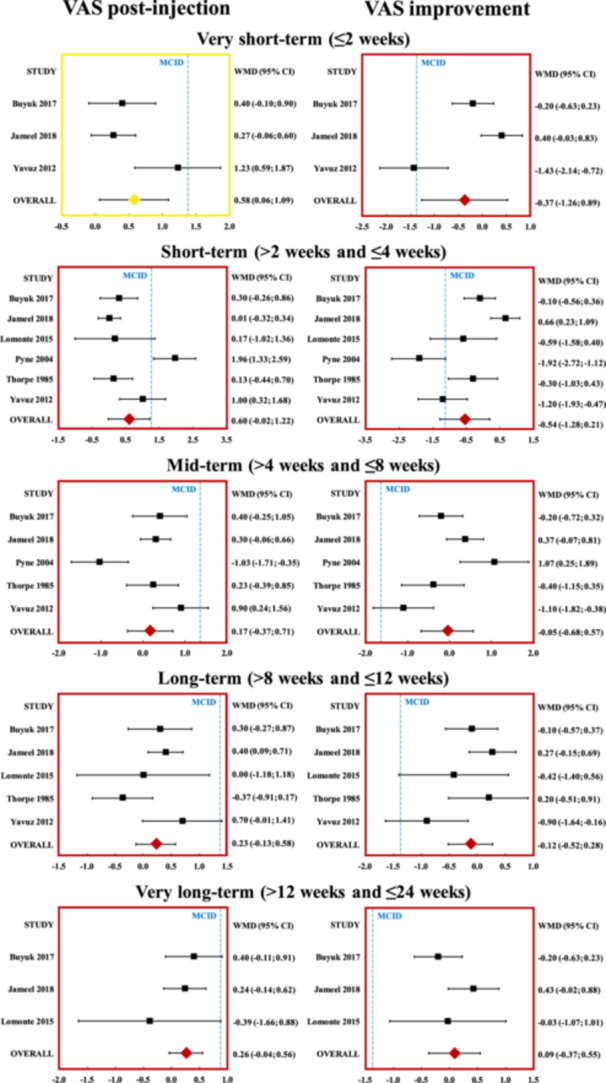
Forest plot of the individual studies and pooled weighted mean difference (MD) for visual analogue scale (VAS) pain post‐injection values and improvements comparing triamcinolone and methylprednisolone, including a 95% confidence interval. Triamcinolone showed better post‐injective VAS values at very short‐term follow‐up, but the MD did not exceed the minimal clinically important difference (MCID) value of 1.37 points. No difference in post‐injection values at other follow‐ups and neither VAS improvement at any follow‐up was found between the two compounds. Red: not statistically significant, yellow: statistically but not clinically significant (MD < MCID).

Among the outcome measures extracted, sufficient data was available to perform a meta‐analysis on the comparison between VAS pain results of triamcinolone and methylprednisolone in knee OA. The meta‐analysis was performed on VAS post‐injection values and improvement compared to baseline at very short‐, short‐, mid‐, long‐, and very long‐term follow‐ups (Figure 3). Based on previous studies, the MCID for VAS pain was set at 1.37 [54].

#### Very short term

The analysis of VAS pain at very short term (≤2 weeks) was performed on three studies, including 256 patients. The meta‐analysis of post‐injection values showed a statistically significant difference in favour of the triamcinolone group (*p* = 0.028, MD = 0.58, SE = 0.26). The MD did not exceed the MCID value of 1.37. The meta‐analysis of VAS improvement from baseline showed no statistically significant difference.

#### Short term

The VAS analysis at short term was performed on six studies, including 371 patients. The meta‐analyses of post‐injection values and improvement from baseline did not show statistically significant differences.

#### Mid term

The VAS analysis at mid term was performed on five studies, including 321 patients. The meta‐analyses of post‐injection values and improvement from baseline did not show statistically significant differences.

#### Long term

The VAS analysis at long term was performed on five studies, including 343 patients. The meta‐analyses of post‐injection values and improvement from baseline did not show statistically significant differences.

#### Very long term

The VAS analysis at very long term was performed on three studies, including 276 patients. The meta‐analyses of post‐injection values and improvement from baseline did not show statistically significant differences.

### Risk of bias and quality of evidence

**Figure 4:**
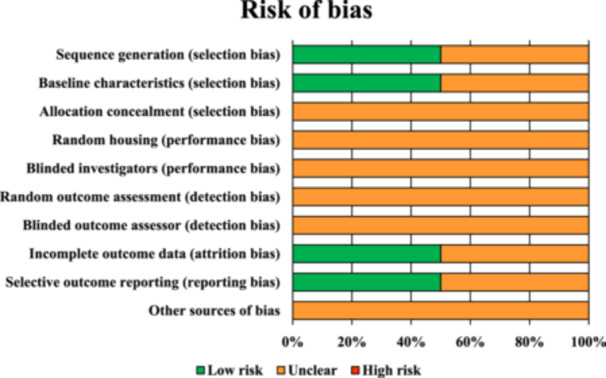
Risk of bias assessments for the included studies according to the SYRCLE's risk of bias tool. The bar chart shows the percentage of all studies that met each quality item, scored as ‘low risk’, ‘high risk’, or ‘unclear’.

A summary of the risk of bias assessment of the two included animal studies according to the SYRCLE tool is illustrated in Figure 4. Most items (80%) were viewed as unclear, while a low risk of bias was observed in the remaining 20% of items. No item was identified as high risk of bias.

**Figure 5:**
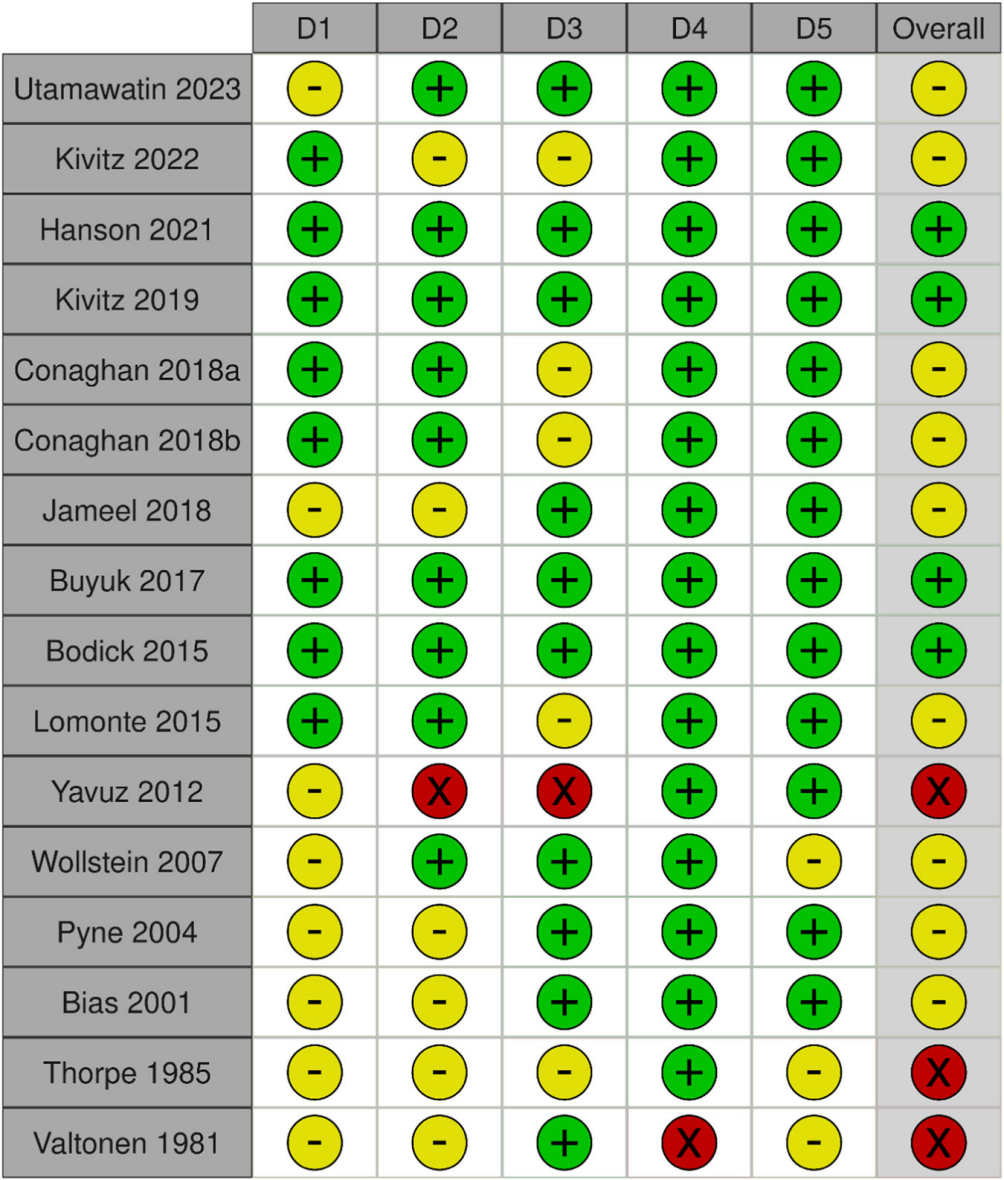
Cochrane risk of bias tool for randomized trials Version 2 (RoB 2.0)—green = low risk; yellow = some concerns; red = high risk.

The evaluation using the RoB 2 tool showed that four studies had a ‘low risk’ of bias, nine studies had ‘some concerns’ and three had a ‘high risk’ of bias. A summary of the risk of bias assessment of the included RCTs is illustrated in Figure 5 [41].

The GRADE evaluation showed that the level of evidence of the results was low for the VAS pain analysis at all follow‐ups. A summary of the quality of evidence assessment of the meta‐analysed outcomes is illustrated in Table 4.

**Table 4:** GRADE evaluation.

Outcomes	Risk of bias	Inconsistency	Indirectness	Imprecision	Publication bias	Other	Quality of the evidence
VAS post‐injection Very short term	Serious	No	No	Serious	No	No	Low ⊕⊕〇〇
VAS post‐injection Short term	Serious	No	No	Serious	No	No	Low ⊕⊕〇〇
VAS post‐injection Mid term	Serious	No	No	Serious	No	No	Low ⊕⊕〇〇
VAS post‐injection Long term	Serious	No	No	Serious	No	No	Low ⊕⊕〇〇
VAS post‐injection Very long term	Serious	No	No	Serious	No	No	Low ⊕⊕〇〇
VAS improvement Very short term	Serious	No	No	Serious	No	No	Low ⊕⊕〇〇
VAS improvement Short term	Serious	No	No	Serious	No	No	Low ⊕⊕〇〇
VAS improvement Mid term	Serious	No	No	Serious	No	No	Low ⊕⊕〇〇
VAS improvement Long term	Serious	No	No	Serious	No	No	Low ⊕⊕〇〇
VAS improvement Very long term	Serious	No	No	Serious	No	No	Low ⊕⊕〇〇

Abbreviation: VAS, visual analogue scale.

## DISCUSSION

The main finding of this systematic review is that the available preclinical and clinical literature provides limited evidence on the comparison of different CS, which hinders the possibility of determining the best approach in terms of CS product and dose for the intra‐articular injection of joints affected by OA. Thus, despite being one of the most widely used injective treatments, it is not possible to provide clear indications for the most suitable CS application for clinical practice.

Intra‐articular CS have been used for decades in the treatment of symptomatic OA, exploiting their anti‐inflammatory and immunomodulatory effects to suppress joint inflammatory processes [7, 15, 55, 63]. Different doses and different products are daily used in clinical practice. It would be paramount to understand the best product and dose to optimize the treatment with intra‐articular CS injections for OA management, both in terms of treatment effectiveness, but also in terms of avoiding the possible detrimental effects attributed to CS. In this regard, most preclinical studies on the effects of CS on articular cartilage were performed on healthy models and reported contrasting results. Nakazawa et al. studied the effects of repeated injections of three different CS types (hydrocortisone acetate, triamcinolone acetonide, dexamethasone acetate), on both in vitro chondrocyte cultures and on a mice model, observing a steroid‐induced arthropathy, with progressive degradation of articular cartilage and joint destruction, as well as the induction of chondrocyte apoptosis in both models following the CS treatment [48]. Similarly, Doyle et al. and Chunekamrai et al. analysed the detrimental effects on equine healthy cartilage of methylprednisolone acetate, administered by intra‐articular injections or to in vitro explants cultures, finding signs of a proteoglycan catabolism induction, chondrocyte necrosis and hypocellularity, loss of proteoglycan content and a marked reduction of collagen synthesis [15, 21]. Severe in vitro toxicity of betamethasone and methylprednisolone has been reported in canine‐explanted chondrocytes and synoviocytes [59]. Multiple studies suggested the detrimental effect of CS on human chondrocyte viability in vitro, with both triamcinolone and methylprednisolone showing chondrotoxic effects, and dexamethasone and even more betamethasone presenting a dose‐dependent effect on cellular necrosis [19, 20, 22, 23, 61]. Overall, the in vitro studies warn against the possible detrimental effects of CS and underline the importance of investigating molecules and doses with the best risk/benefit ratio to treat OA joints.

A number of controlled in vivo studies have been performed as well, including the large animal model in the horse, to both clarify the therapeutic response as well as the possible deleterious effects of intra‐articular CS products like triamcinolone acetonide, methylprednisolone acetate and betamethasone esters [42, 43]. Two studies were performed using an equine chip fragment model of OA and the most significant results were both symptom‐modifying and disease‐modifying effects with injections of 12 mg of triamcinolone acetonide 2 and 4 weeks after induction of OA [25]. In contrast, methylprednisolone acetate (100 mg dose at 2 and 4 weeks) produced variable symptom‐modifying effects but, rather than significant beneficial disease‐modifying effects, produced significant deleterious effects on the articular cartilage [24]. Accordingly, because of the critical nature of welfare issues in equine athletic competition, the use of methylprednisolone has been stopped in racehorses (controlled by drug testing). This shows the importance of preclinical studies in driving indication for CS use. Unfortunately, while the analysis of the animal evidence could provide the proper analyses to document the tissue changes induced by CS in OA joints, this systematic review revealed a remarkably limited number of studies exploring the different effects induced by CS molecules and doses. In fact, only two RCTs were retrieved, with one study addressing the comparison of different CS and another study analysing the results of different doses of the same compound in animal OA models. The study of Alves et al. compared triamcinolone and methylprednisolone reporting overall similar clinical results of the two compounds in naturally occurring hip OA in dogs [1]. The study of Williams et al. analysed the effects of different doses of triamcinolone in knee OA induced in guinea pigs, showing a positive dose‐dependent effect on articular cartilage [69].

More data emerged from the analysis of the clinical evidence on different CS types and doses for OA management. Knee was by far the most studied joint, with 14 RCTs, while two RCTs addressed shoulder OA and only one RCT hip OA. This distribution reveals a considerable paucity of literature analysing joints other than the knee, hindering the possibility of drawing solid conclusions on the results obtained in different joints by CS injections, as the optimal CS type and dose may be different depending on the joint treated. In clinical practice, these aspects are frequently left at the discretion of the clinician: in fact, there is a lack of standardization of CS injection procedures not only across the world but also within the same countries, which underpins the arbitrary use of CS preparations at different anatomical locations [13, 58]. In this scenario, there is an urgent need for more standardized procedures and the identification of the best CS type and dose for each joint to maximize CS treatment efficacy and optimize the management of OA in clinical practice.

A large variety of CS is available on the market, including dexamethasone, betamethasone, hydrocortisone, prednisolone, triamcinolone, and methylprednisolone, the last two being the most frequently used for intra‐articular injection in clinical practice [56, 64]. Among these, triamcinolone, methylprednisolone, and betamethasone emerged as the options investigated by the current literature in high‐level clinical trials addressing the comparison between different intra‐articular CS for OA management. Triamcinolone and methylprednisolone were studied in 14 and 6 RCTs, respectively, reflecting the trend of use of the available CS in the clinical practice, while betamethasone was analysed by only 3 RCTs. In particular, the study of Wollstein et al. reported equivalent pain relief up to three weeks after betamethasone and methylprednisolone injection in knee and shoulder OA [70]. However, this study did not separate the results of the two compounds for the two considered joints, creating an important confounder for data analysis and interpretation. More recently, Yavuz et al. reported better results of methylprednisolone compared to betamethasone in terms of pain relief up to three weeks after injection, but not in terms of functional improvement [71]. The only study comparing betamethasone with triamcinolone by Valtonen et al. showed that the latter was able to provide superior pain and joint tenderness reduction one week after injection in knee OA patients [67]. Studies on a larger number of patients are needed to confirm the suggested superiority of the two most used CS compared to betamethasone as well as to investigate the comparison with other alternative compounds, also stratifying the results according to the joints treated.

Triamcinolone and methylprednisolone were the most investigated compounds. In particular, the knee joint presented a sufficient number of RCTs with VAS pain having a suitable amount of data to perform a level I meta‐analysis. The results showed no difference in terms of pain improvement between the two. In fact, while triamcinolone showed better post‐injection VAS values only at very short‐term follow‐up, this difference did not reach the MCID threshold, failing to prove clinical significance for patients affected by knee OA. Overall, these findings support the equivalence of triamcinolone and methylprednisolone when used as an injective therapy to address knee OA. Still, these analyses were performed on a limited number of patients, ranging from only 256 to 371 at the different follow‐ups, and more data are needed to confirm both the potential and safety profile of these two products and understand if they are really equivalent for clinical use, or if one compound may prove more suitable to treat OA.

In all the studies included in this meta‐analysis against methylprednisolone, triamcinolone acetonide was used in the traditional crystalline suspension formulation. However, an emerging formulation currently gaining attention in the field of CS injections is represented by extended‐release triamcinolone acetonide. This product is formulated in poly (lactic‐co‐glycolic acid) microspheres that slowly release triamcinolone acetonide in the synovium, to prolong its presence in the joint while reducing systemic exposure and adverse events [51]. Five of the RCTs included in this study addressed the comparison between the crystalline suspension and the extended‐release formulations of triamcinolone acetonide, with three RCTs performing this comparison in knee OA, one in hip OA, and one in shoulder OA. All studies showed the equivalence of the safety profiles of the two formulations. Three studies evaluated the systemic exposure in terms of plasma triamcinolone acetonide concentration, all showing lower values for the extended‐release formulation. Two RCTs investigated the clinical effectiveness of the two formulations: both studies reported superior functional benefits for the extended‐release formulation up to 12 weeks after injection, while discordant results were reported for VAS pain improvement. Of note, both these studies were sponsored, creating a potential commercial bias, and independent studies are needed to confirm these results and determine the optimal formulation of triamcinolone acetonide for intra‐articular injection to manage OA.

Another crucial aspect in the field of intra‐articular CS injections is represented by the dose of the CS administered. Four RCTs investigated the outcomes of different doses of CS in knee OA. Two studies compared the results of different doses of triamcinolone acetonide extended‐release, both suggesting a moderate dose‐response correlation (16 mg vs. 32 mg and 10 mg vs. 40 mg, respectively). Another RCT compared triamcinolone acetonide crystalline suspension 10 mg versus 40 mg reporting similar pain relief for the two doses. An RCT focused on dexamethasone palmitate comparing 4 mg versus 12 mg doses, but it was not blinded and did not perform statistical analysis on the clinical outcomes, hindering the possibility of drawing reliable conclusions. Overall, a limited amount of data is currently available in the literature regarding the optimal CS dose and future high‐level studies should aim at investigating this important aspect of intra‐articular CS injections for OA.

Safety represents another key aspect of the comparison between different CS for OA injective treatment. While intra‐articular CS injections are relatively safe and present a low risk of short‐term complications [31, 39], concerns have been raised about their long‐term effects on articular cartilage, with some studies reporting negative effects, especially after repeated administration [40, 72]. However, no decisive evidence is currently available on the superiority of a compound over the others in terms of both short‐ and long‐term safety profile. Overall, many minor adverse events were reported, but only a few unrelated more serious events. Especially in terms of safety profiles, more data are needed to compare CS products. Moreover, specific evaluations should be performed to elucidate the effects at the joint level, either by exploring biomarkers of tissue degradation or by documenting degenerative changes with imaging evaluations. In fact, a possible detrimental effect of CS has been documented when investigating patients treated with CS at 2 years of follow‐up. In a recent saline‐controlled, double‐blind RCT on 140 knee OA patients receiving an intra‐articular injection of 40 mg of triamcinolone every 3 months, McAlindon et al. performed a magnetic resonance imaging evaluation revealing a significantly greater cartilage volume loss in patients treated with CS injections compared to saline controls [40]. A study from the Osteoarthritis Initiative of Zeng et al. confirmed that intra‐articular CS injections, especially repeated CS administrations, are associated with an increased risk of knee OA progression in terms of KL grade and joint space narrowing compared to controls [72]. Future high‐level trials should also compare the potential difference in terms of detrimental effects on the articular surface of different compounds and doses at longer follow‐ups, to optimize the choice of CS among the available options for OA injections in the clinical practice.

The available literature on the CS comparison revealed a limited number of studies with an overall limited quality. Thus, the limitations of the present study reflect the limitations of the available literature. First, the low amount of data on CS other than triamcinolone and methylprednisolone hindered the possibility of performing a meta‐analysis on the outcomes of different compounds. Similarly, not enough data were available to meta‐analyse the results of different doses of the same CS nor the results of different formulations of triamcinolone acetonide. Additionally, the selected RCTs lacked standardization in terms of data collection (particularly patients' age, BMI, associated treatments, and other basal characteristics being not homogeneous or not reported in many of the included articles) and reporting of outcome measures, adverse events, and associated follow‐up timeframes, not allowing to reach clear conclusions as well as to perform a meta‐analysis on outcomes different from VAS for pain. Moreover, there is poor evidence of the effects of intra‐articular CS injections in different anatomical districts, with the wide majority of studies addressing knee OA, making it difficult to draw reliable conclusions on the possible different effects of CS injections on different joints. Finally, some of the studies received commercial funding, creating a potential commercial bias.

Despite these limitations, the present systematic review and meta‐analysis provided a thorough investigation of the available literature on the comparison of different CS types and doses to manage OA, demonstrating the equivalence of triamcinolone and methylprednisolone for knee OA injection. Future studies should aim at investigating the comparison with other alternative CS, also stratifying the results according to the joints treated, as well as at determining the optimal dose and formulation of the available compounds for the treatment of joints affected by OA.

## CONCLUSION

The available preclinical and clinical literature provides limited evidence on the comparison of different CS, which hinders the possibility of determining the best CS approach in terms of molecule and dose for the intra‐articular injection of joints affected by OA. Thus, despite being one of the most widely used injective treatments, it is not possible to provide clear indications for the most suitable CS application for clinical practice.

## AUTHOR CONTRIBUTIONS

Conceptualization: Giuseppe Filardo. Methodology: Alessandro Bensa, Manuela Salerno, and Giacomo Moraca. Data Curation: Alessandro Bensa and Angelo Boffa. Writing—original draft preparation: Alessandro Bensa, Manuela Salerno, Giacomo Moraca, and Angelo Boffa. Writing—review and editing: C. Wayne McIlwraith and Giuseppe Filardo. Supervision: Giuseppe Filardo. All authors have read and agreed to the published version of the manuscript.

## FUNDING INFORMATION

This research did not receive any specific grant from any funding agency in the public, commercial or not‐for‐profit sectors.

## CONFLICT OF INTEREST STATEMENT

The authors declare no conflict of interest.

## ETHICS STATEMENT

The ethics statement is not available.

## Data Availability

All studies included are publicly available. Our analysis can be shared upon request.
